# Mobile Phones in a Traffic Flow: A Geographical Perspective to Evening Rush Hour Traffic Analysis Using Call Detail Records

**DOI:** 10.1371/journal.pone.0049171

**Published:** 2012-11-14

**Authors:** Olle Järv, Rein Ahas, Erki Saluveer, Ben Derudder, Frank Witlox

**Affiliations:** 1 Department of Geography, University of Tartu, Tartu, Estonia; 2 Department of Geography, Ghent University, Ghent, Belgium; 3 Positium LBS, Tartu, Estonia; University of Namur, Belgium

## Abstract

Excessive land use and suburbanisation around densely populated urban areas has gone hand in hand with a growth in overall transportation and discussions about causality of traffic congestions. The objective of this paper is to gain new insight regarding the composition of traffic flows, and to reveal how and to what extent suburbanites’ travelling affects rush hour traffic. We put forward an alternative methodological approach using call detail records of mobile phones to assess the composition of traffic flows during the evening rush hour in Tallinn, Estonia. We found that daily commuting and suburbanites influence transportation demand by amplifying the evening rush hour traffic, although daily commuting trips comprises only 31% of all movement at that time. The geography of the Friday evening rush hour is distinctive from other working days, presumably in connection with domestic tourism and leisure time activities. This suggests that the rise of the overall mobility of individuals due to societal changes may play a greater role in evening rush hour traffic conditions than does the impact of suburbanisation.

## Introduction

It is a common understanding that suburbanisation and urban sprawl in particular is deemed responsible for increasing traffic and rush hour congestions [1]. This assertion is convincing, as recent extensive land use changes around densely populated urban areas has gone hand in hand with a growth in transportation demand. Nevertheless, the relationship between suburban land use and transportation, as well as its magnitude has remained somewhat unclear due to the controversial nature of study findings [2]–[8]. The relationship is not simple as it first seems and thus, transport demand must be understood in the wider context of urban dynamics and society [9] while it is caused by countless processes and underlying factors with their complex interrelationships [10]–[13].

### Factors Driving Transportation Demand

According to Pred [14], combining Giddens’ structuration theory [15] with the concept of time geography developed by Hägerstrand [16], developments within societies and their fundamental structures have and are changing human actors and their everyday life practices. Vice versa, through individuals’ daily practice people intentionally or unintentionally produce and reproduce societal structures and systems. The interplay of the individual and society directly or indirectly continuously affect individual activity travel behaviour and transportation needs. Currently, societal structures in post-industrial societies are changing due to the overall growth in prosperity and the shift in the labour market, globalization and development of mobile technologies. This affects individuals’ everyday practices, their needs and lifestyles due to flexible working schedules and increasing leisure time [17], [18], the increase of car dependency [19], the adoption of information and telecommunication technologies (ICT) [20]–[22] and the growing importance of social networks [23], [24]. Due to a constant increase of the movement of people, goods and information all over the globe, Sheller & Urry [25] argue that we are dealing with a “new mobilities paradigm” in the 21^st^ century. Although mobility is seen as both a spatial and social phenomenon [26], we focus on mobility as the physical movement in space while containing a social meaning.

With growing mobility of individuals, there is a need for solving issues related to increasing transportation volumes and individual transportation needs that have a negative impact on society – e.g. congestion, dissatisfaction with the quality of life, CO_2_ pollution, excessive use of natural resources [27]–[29]. The effective intervention of traffic congestion is challenging, hence the phenomenon is both physical and psychological [10], [30] and the phenomenon is difficult to unfold due to the complexity of direct, indirect and random factors [30]. Direct factors (mode split, drivers’ behaviour, road design) affect at a micro scale, on the road, and are seen as “triggers” for traffic congestion [31]. Indirect factors are underlying factors derived from a society that influence the overall transportation demand and thus, contribute to the nature of congestion and to its magnitude [27], [32], [33]. Besides underlying societal factors, e.g. layout of urban structure, demographics, infrastructure, socioeconomics or legislation, travel behaviour of individuals is also shaped by cultural habits and societal norms [34] along with human actors themselves with their attitudes, socioeconomic variables, life quality values or lifestyles [35]–[40].

Transport demand is, until now, considered predominantly as a derived demand [41] and hence, traffic flows are broadly divided into three categories by trip purposes – mandatory or subsistence (work or school-related), maintenance (shopping, personal- or household-related), and leisure [42], [43]. Both in Europe as well as in North-America, work-related or mandatory travel constitutes approximately one fifth of all trips which people make and up to one fourth of all their distance travelled whereas both measures have decreasing trend [5], [44], [45]. On the contrary, the biggest share of trips and person-kilometres with an increasing trend are travelled for leisure purposes. Furthermore, due to changes in society individuals’ activity travel behaviour have become more flexible, fragmented, mobility- and adventure-seeking [46], and thus their objective may well be travel itself as an activity [47], [25]. During rush hour, however, Downs [26] claims that roughly half of the trips are work-related, although, trip chaining and stop-making behaviour during the work commute makes it difficult to distinguish mandatory and non-work related trips [48] in order, for example, to assess work-related suburban commuting [49].

### Methodological Approaches for Studying Traffic Flows

Although transportation and congestion are thoroughly studied [28], [50] and a variety of measures are used to steer transportation demand along with mitigation of traffic congestion [42], [51], [52], the latter deal with the consequences. In order to better understand underlying causes for traffic concentration problems and to find solutions, different intelligent transportation systems (ITS) have been developed. ITS have already become one effective tactic for the alleviation of worsening traffic conditions, however, the effectiveness can be improved further by implementing different applications of ICT. For example, mobile phone positioning data is proposed and applied as one promising movement data collecting method, providing advantages over traditional methods of traffic measurement (e.g. road-survey questionnaires, traffic counters, travel diaries) [53]–[56]. It is more cost-effective in the context of continuous macro-scale mobility studies than conventional questionnaires or travel diaries, and thus it enables the provision of a large amount of data over long study periods, coverage of large study areas with unlimited sample size potential, and in addition data can be processed into movement information in real time. However, it has some disadvantages such as privacy issues, access to data and sampling issues related to phone ownership and its use.

Considering the advantages and given the inevitable ubiquity of mobile phones in our daily lives [57], [58], mobile phone data is able to offer a better understanding of individuals’ mobility patterns within a society from a temporal and geographical perspective as a valuable complement to traditional methods in transportation studies [59]–[64]. Prior to this study our workgroup applied the same data and similar approach while investigating the general composition of traffic flows on the three main highways for assessing the impact of investment on infrastructure in Estonia [65]. The comparison of traffic flows between extracted mobile phone data and the stationary traffic counter as well as the comparison of origin-destination matrices between extracted mobile phone data and the on-road survey with the help of police coincided well and gave us confidence to apply mobile phone data in transportation studies.

### Research Objectives

This paper aims to contribute to this line of research by putting forward an alternative methodological approach for studying traffic flows using individual mobile phone positioning data [66]–[68]. Thus, the objective of this paper is twofold: (i) to propose an alternative methodological approach that enables to identify the composition of traffic flow and road users’ trip purposes using call detail records of mobile phones; and (ii) to gain new insight regarding the composition of traffic flow and the impact of suburbanites during afternoon rush hour traffic flow on a major highway entering Tallinn, the capital of Estonia. This approach can be applied in real-time and produces valuable data implementation in the framework of ITS applications to monitor traffic flows for effective planning and managing the road network.

Next, we provide the details regarding our proposed approach with the study design – the specification of applied data and method, and background assumptions. Attention then turns towards empirical results, where we first investigate the composition of traffic flow with the geographical distribution of road users by their home and workplace locations in order to assess the origin of road users, and how the distribution changes in time. Further, we assess road users’ trip indication and how it changes with time by linking road user’s home and workplace locations based on the shortest path. Lastly, to assess the differences of trip purposes on Friday’s we compare road users’ geographical distribution of the final destination of the day between Friday’s and other working days. Empirical results are followed by a discussion about research findings.

The study is relevant for receiving new insight regarding the impact of suburbanites on traffic in Tallinn while the suburbanisation process in a post-communist city differs from the classical western model [69], [70]. Moreover, substantial investments on transportation infrastructure in Estonia are currently made in order to solve concentrations of transport demand with the support of European Union cohesion funds.

## Data and Methods

### Study Area

**Figure 1:**
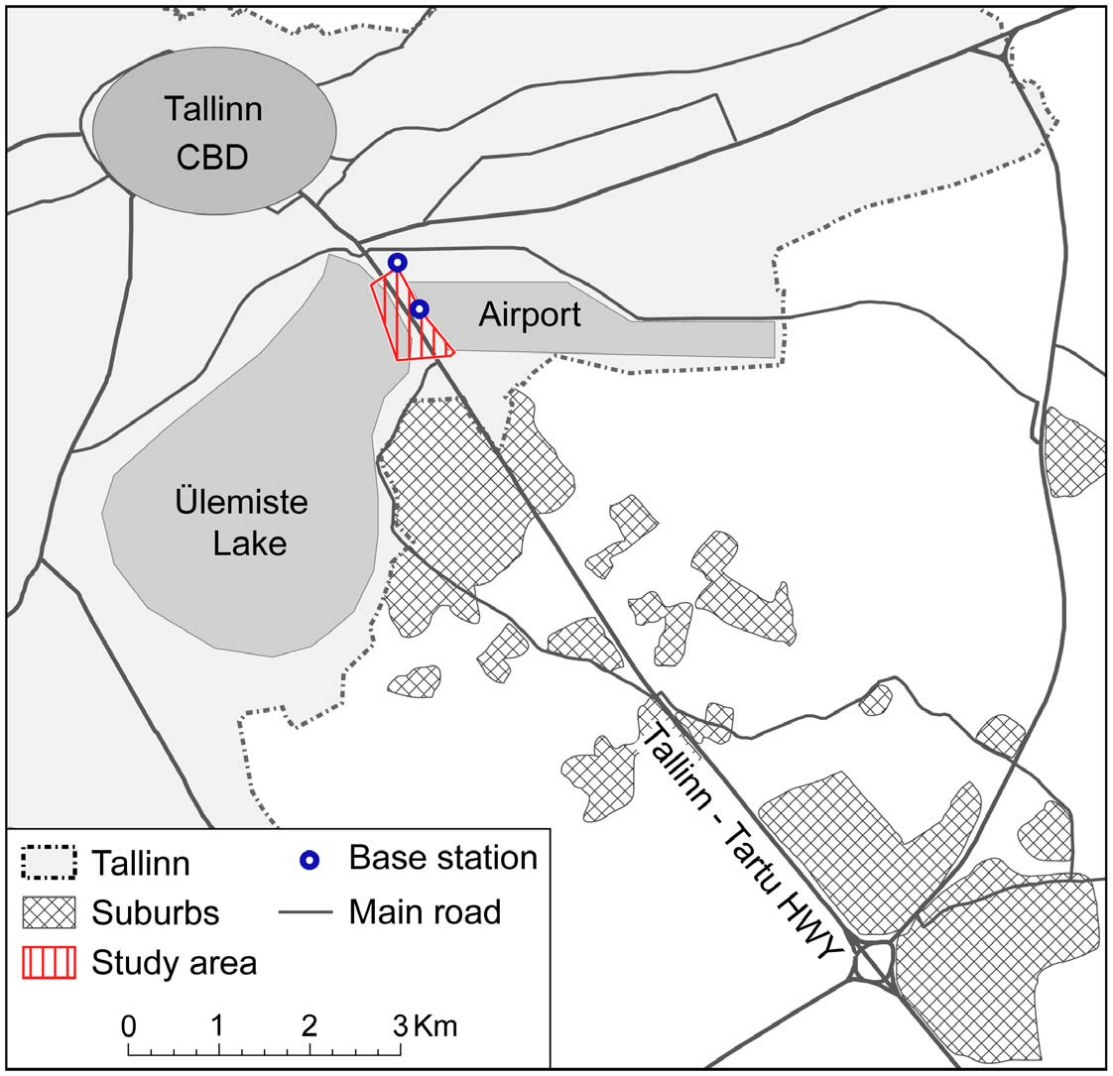
The studied road section along the Tallinn-Tartu highway and its surrounding area. The studied road section is located on the city’s administrative border and surrounded by Ülemiste Lake on the west side and by the airport on the east side of the highway, creating a bottleneck situation for the road network. The vicinity of the Tallinn–Tartu highway within the Tallinn FUR is characterized by rural municipalities where new (sub)urban settlements have recently been developed: commercial and industrial areas along the highway and sparsely located small residential settlements.

Our study area is located in the Tallinn functional urban region (FUR) – the largest FUR and capital city region of Estonia, with approximately 550,000 inhabitants, of whom 400,000 live in the core city of Tallinn (see Text S1 for detail description). We studied a 1.5 km long road section of the Tallinn-Tartu highway (E263) in Tallinn (Figure 1). The highway connects the capital Tallinn and Estonia’s second largest city Tartu (98,473 inhabitants), and is therefore one of the busiest roads in the whole country.

**Figure 2:**
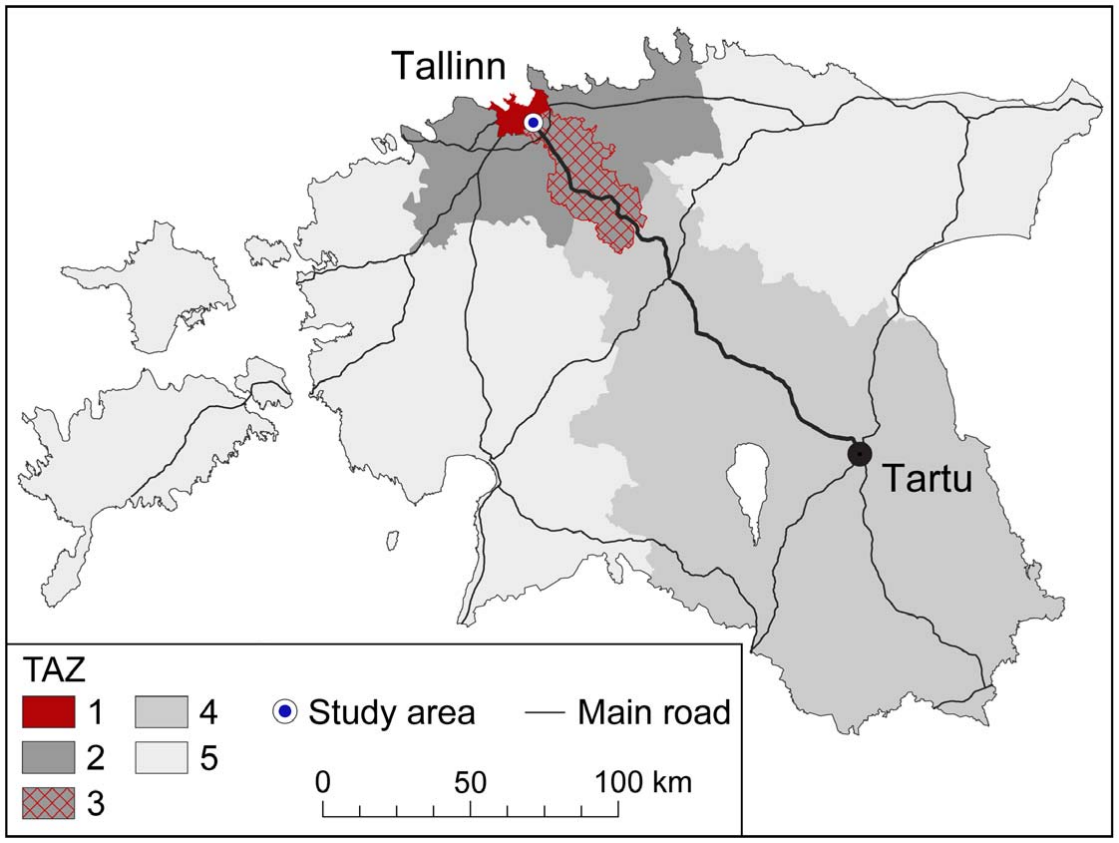
Estonia, aggregated into traffic analysis zones (TAZ) due to relative location to the study area. Description of TAZs is as follows: TAZ 1 (Tallinn), the capital of Estonia and the centre of the Tallinn FUR; TAZ 2 (FUR), the functional urban region of Tallinn includes all municipalities where at least 15 per cent of the workforce has its workplace in Tallinn, and excludes the catchment area of the Tallinn-Tartu highway; TAZ 3 (FUR, catchment area), the part of the Tallinn FUR that due to its relative location to the study area on the Tallinn-Tartu highway is considered to be the shortest and fastest highway connection to Tallinn for local inhabitants; TAZ 4 (Estonia, catchment area), the part of Estonia that is outside the Tallinn FUR and which due to its relative location to the study area is considered to be the shortest and fastest highway connection to Tallinn; TAZ 5 (Estonia, other), Western and North-Eastern Estonia, which is outside of the Tallinn FUR and not directly connected to the Tallinn-Tartu highway catchment area.

The studied road section is covered by four antennas of two mobile network base stations, and thus the coverage area of this operator’s antennas will be considered as the study area (Figure 1). For analytical purposes, Estonia was aggregated into five traffic analysis zones (TAZ) depending on its relative location to the study area (Figure 2).

### Data Description

Nearly 95% of Estonia’s 1.38 million inhabitants used mobile phones during the study period [71]. We use the mobile network operator’s call detail records (CDR) of mobile phones which is also known as passive mobile phone positioning data [61]. CDR originates from the largest Estonian mobile network operator EMT, which has over half a million active clients distributed throughout Estonia [71]. Data is further processed by a spin-off company Positium LBS. The study period was one year, from October 1^st^ 2008 to September 30^th^ 2009, and all of EMT’s active clients were included in the analyses. The database consists of records of all outgoing call activities such as calls, SMSs, internet and data services initiated by the phone owner. Each record (i.e. outgoing call activity) in the database includes spatial and temporal parameters (Table 1).

**Table 1:** A random extract from the raw database of call detail records.

FID	User ID	Date	Time	Call activity	Base station ID	X	Y
10001	563371	02.03.2009	10:34:24	Call	545	585934.95	6606320.83
10002	238842	02.03.2009	10:35:06	Call	198	422253.90	6518901.60
10003	238842	02.03.2009	10:41:12	SMS	344	527933.09	6567106.66

Each record includes: the random ID number of the phone (not related to the phone or SIM card number); the exact time and date of the call activity; a geographical location which is determined by the precision of a mobile network antenna (Cell ID) that provides the network signal for a call activity.

Data is encrypted in order to preserve the anonymity and privacy of the studied mobile phone users, and the random identification code is generated by the network operator for every mobile phone to link call activities made by one person (mobile phone) during the study period. Data is recorded in accordance with the Estonian legislation for billing purposes by the network operator and not for the purpose of this study. Data receiving, storage, processes and applications in this research observe data security and privacy requirements specified in EU directives on handling personal data and the protection of privacy in the electronic communications sector [72]. Prior to conducting our research, we have consulted ethical and privacy issues and our approach is approved both by the Estonian State Data Protection Agency and by the Ethical Committee of Human Studies of the University of Tartu. Subscribers of the present network operator are aware of possible data use for scientific research through public relations work: since 2004, the network operator and the research team from the University of Tartu have released several public announcements via major newspapers and television related to research using mobile phone data in Estonia.

Geographical information is obtained based on the geographical coordinates of every network operator’s network antenna and the precision of spatial accuracy of call activities corresponds to the coverage area of a network antenna. A coverage area is not spatially fixed and varies predominately depending on population density, i.e. the use of the mobile phone network. The average size of a network antenna coverage area in densely inhabited Tallinn is 0.8 km^2^, in the Tallinn FUR it is 15.3 km^2^, and in less populated rural areas approximately 120 km^2^.

The road users of the study area were studied in the afternoon period of six hours (2:00–8:00 pm) during 12 months in 2009. Special attention is given to the evening rush hour (4:00–6:00 pm) although hourly differences in the distribution of road users are compared. The results were compared between Friday and other working days (average for Monday – Thursday).

### Factors Influencing the Use of Mobile Phones

In dealing with call activities made by mobile phone users one has to bear in mind that the distribution of call activities is strongly affected by diurnal as well as weekly rhythms of society. Further, several other factors may influence mobile phone use which has to be taken into account. First, the use of mobile phones depends on national context and cultural background – e.g. legislation, religion, moral and ethics, laws concerning phone use, domestication of mobile phones, network availability and service cost [58], [73]. In the context of this study (Estonia), mobile devices are well introduced regardless of socio-economic background and mobile devices are explicitly preferred to landline phones [74]. According to Statistics Estonia [75] the penetration of mobile phone subscriptions (per 100 inhabitants) in Estonia is 126% and 1.7 billion calls were made with mobile phones in comparison to 0.2 billion calls through landline phones during the study period. Secondly, if in certain places the use of a mobile phone may be partly or fully restricted (e.g. courthouse, library), nevertheless a digital footprint can be recorded. One can make call activities at these places prior to or after a certain activity is performed, and in case voice calls are forbidden other means such as SMS or web services can be used instead. Thirdly, making call activities varies in space and time among individuals by their socio-economic characteristics as well as attitudes, self preferences, lifestyles, habits and work attributes [58]. The anchor point model we implement [68] takes into account spatio-temporal variance of mobile phone usage and reduces errors due to this matter. Fourthly, smartphones are adopted at a phenomenal pace and are being embedded into our daily lives due to their use throughout the day simultaneously while conducting other activities [76]. For example, 87% of smartphone owners use their phone while they are on-the-go (e.g. commuting, walking) [77]. Thus, smartphones will provide a more precise digital footprint (based on outgoing calls) including places where physical activities are conducted as well as on the move due to comprehensive data transfers. At the time of the study smartphones had a smaller impact due to a lower penetration, however, to date in developed countries the penetration is reaching a level of 50% [76], [78] and the current trend of adapting smartphones around the world will improve the quality and precision of analysis based on a digital footprint via mobile phones.

Finally, the use of mobile devices while driving is being banned by the law or restricted to only the use of hands-free devices with a slow pace [79]. Despite that the use of a mobile phone while driving is legally prohibited and recognized by people as a dangerous act in several countries, it is still practiced [80], [81]. In our case, the use of mobile devices while driving is commonly practiced in Estonia and was restricted by the law only in urban areas allowing the use of a hands-free appliance. Thus, we assume that our sample includes all non-driving passengers who were using a mobile phone, all drivers using a hands-free device and all drivers who illegally used a hand-held mobile phone while driving.

### Background Assumptions and Method Description

We made three assumptions regarding biased mobile phone usage and the confidentiality of the results. First, call activities made within the study area represent a random sample of all road users and are thus sufficiently representative to generalise the whole traffic flow in a given road section. Second, we assume call activities that are made during a one month period is long enough to reflect a person’s monthly activity space which is a construction from habitual routine and variety seeking behaviour [82]. Third, although mobile phone usage is biased by several factors as explained above, we assume that these possible biases are spatially and temporally equivalent and thus do not affect the study results from a geographical or temporal perspective.

We conducted our research as follows. We selected and analysed all mobile phone users who made at least one call activity from one of four studied network antennas during the study period. We located the two most important everyday anchor points in their activity space (i.e. home and work-time location) by using the anchor point model based on their mobile phone call activity data [68]. Several studies have adapted mobile phone call activity data for identifying individual’s important activity places [62], [83]–[87]. According to Ahas *et al*. [68] the anchor point model in general, finds for every month the two most frequently used mobile antennas where call activities are made as everyday anchor points, and further, the algorithm distinguishes everyday anchor points into home and work-time (work, or school-related) locations taking into account (i) the average of (outgoing) calling times and (ii) the standard deviation, and (iii) spatial neighbouring relationships of anchor points (see [68], pages 11–16 for further details). Hereinafter, we use “workplace location” as a synonym for a “work-time location” which is proposed by Ahas *et al*. [68] and denotes the most frequently visited place during office hours by a person besides the home location. The anchor point model [68] as well as other similar approaches [88] has obtained a high reliability in verifying the correctness of the algorithm with real home and workplace locations of individuals. With high reliability of the anchor point model and while the results of the model are representative of the adult population of the country [68] we are, therefore, confident that the model is suitable for the current research.

For the movement analysis, every road user’s (mobile phone user) locations of everyday anchor points (workplace and home) were identified, linked and transformed into a workplace-home movement vector which was downscaled to the roadway network. According to the shortest path analysis road users were classified by their home and workplace location into commuters and into other (non-commuter) road users. A commuter is defined as a road user whose home and workplace anchor points are located in separate municipalities and which, according to the shortest path analysis, most probably uses the studied road section for home-workplace-home movement. Commuting is assigned whenever a road user who is defined as a commuter uses his/her phone in a studied road section, i.e. when a commuter uses a mobile phone in the studied road section we define the movement as commuting. Note that we do not know 100% whether it is actually work-related commuting or leisure travel (if suburban commuter has a day off from work and uses their everyday route to return from shopping purposes instead), and in this sense we consider “commuting” as every movement in the studied road section. However, given the time span of the collected data (evening peak hours), and the methodology used we may assume that commuters’ traffic movement during evening peak hours is predominantly a commuting trip from workplace to home. The use of a mobile phone by other (non-commuter) road users is assigned as “other non-commuting” movement. To some extent the latter movement can include non-direct (trip-chaining) workplace-home movements, although considering the spatial context in our study area, this should be marginal (see Figure 1).

The final destination of the day for road users passing the studied road section during the evening rush hour was defined as the locations of the last call activity at the end of the day. The distance between the final destination and home location is calculated using the shortest path analysis. The end of the day was set at 4:00 am of the next day, thus some movements ended in the first hours of the next day.

### Traffic Flow in Study Area

In order to implement mobile phone data for analysing hourly traffic flows, one must first eliminate diurnal and weekly rhythms of the call activity pattern. Thus we calibrated our modelled movement data with the nearest stationary loop detector traffic counter data which is located on the Tallinn-Tartu highway (E263) near the administrative border of Tallinn provided by the Estonian Road Administration [89]. To this end, first, we used the simple linear regression analysis to fit a daily total call activity data of each weekday with the daily total traffic count in order to eliminate a weekly pattern of call activities. Hence, we found a regression model for each weekday. Further, we used a simple linear regression analysis to fit hourly call activity data with the hourly traffic count. Hence, we found a regression model for every hour of the day separately for working days (Monday – Friday), Saturdays and Sundays.

**Figure 3:**
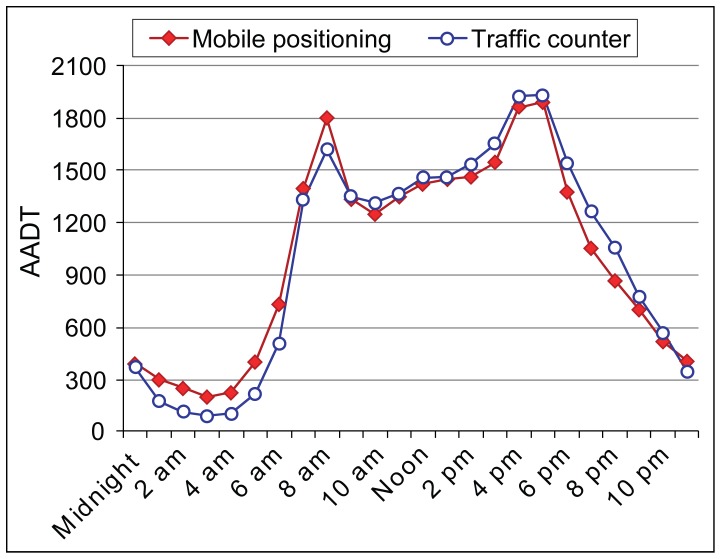
The comparison of AADT between the traffic counter and mobile phone data. The comparison of annual average daily traffic (AADT) between the stationary traffic counter data and estimated traffic flow extracted from the calibrated mobile phone data by hourly frequencies during the study period.

After calibration, the correlation between the annual average daily traffic (AADT) from the traffic counter and our modelled traffic flow from mobile phone positioning data was very high, R^2^ = 0.98 (Figure 3). In other words, mobile phone data, i.e. call detail records of mobile phones seem to capture rather well the traffic flows intensities as compared to conventional traffic counter data. AADT generated from mobile phone data amounted to 24,313 (SD 5,328) vehicles for both ways, while with the stationary traffic counter it amounted to 24,420 (SD 4,589) vehicles [89]. The greatest differences between the two datasets occurred during the night (midnight –6:00 am), when mobile positioning data overestimates flows, and during the evening (6:00 pm –9:00 pm), when it underestimates flows. For this study, we assume these differences are not relevant as this kind of possible systematic error, if present, would not affect the composition of road users by their geographical distribution of home and workplace locations.

**Figure 4:**
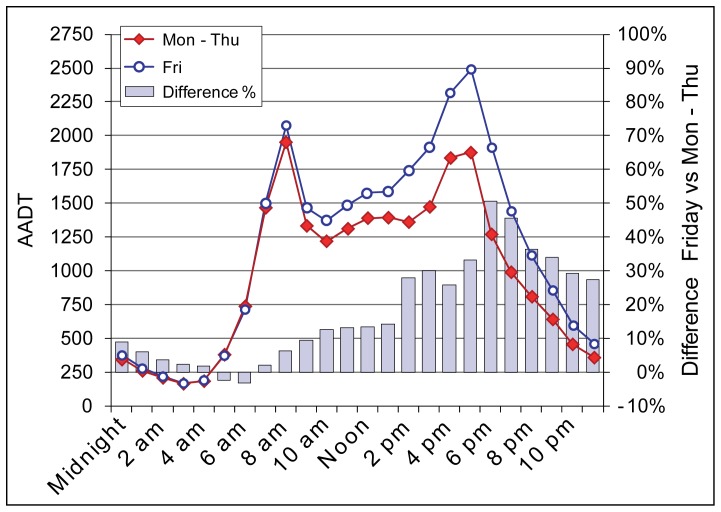
The comparison of AADT between Friday’s and other working days. The comparison of annual average daily traffic (AADT) between Friday’s and other working days by hourly frequencies during the study period. AADT is based on mobile phone data.

From Mondays to Thursdays the average daily traffic is 12% higher than AADT, while on Friday’s the average daily traffic is 35% higher than AADT. Traffic during the morning and evening rush hour peaks from Monday to Thursday is equal (around 1600 vehicles/hour) (Figure 4). On the other hand, traffic during the Friday evening (2:00 pm –6:00 pm) is most intense of the whole week. When comparing hourly traffic on Friday with other working days (the average from Monday to Thursday), the traffic remains denser from the morning rush hour (+10%), and increases rapidly two hours prior to the evening rush hour (Figure 4, see columns).

The difference on Friday compared to other working days prior to and during the rush hour is some 30% higher. Immediately following the evening rush hour, traffic on Friday is up to 50% higher, which clearly extends the rush hour by one hour, whereas before the end of the day traffic remains some 30% higher than on other working days. These previous aspects of the Friday evening traffic clearly shows the difference compared to a typical working day. In the following sections we demonstrate the composition of traffic flow and how it changes in the afternoon in various aspects.

## Results

### Distribution of Road Users by Home and Workplace Location

**Figure 5:**
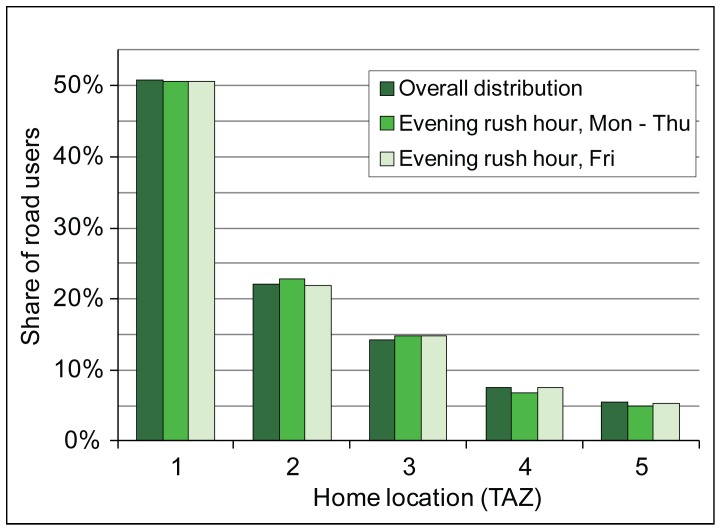
Distribution of road users by home locations. T he distribution of home locations located in five transport analysis zones (TAZ, see Figure 2) is shown separately for the annual overall distribution and during the evening rush hour on Friday’s and on other working days.

The annual average distribution of road users by their home location in the studied road section indicates that half of them have their home location in the city of Tallinn (TAZ 1, Figure 5). Road users who live in a part of the Tallinn FUR that, due to its relative location is considered to be a catchment area for the studied road section (TAZ 3), comprise 14% of all road users. In other words, every seventh road user is considered to be a suburbanite who most probably uses the studied road on a daily basis. The remainder of road users living in the Tallinn FUR (TAZ 2) comprise 22% of all road users. Road users who live outside the Tallinn FUR comprise 14% of all road users. We found no statistically significant differences in the composition of home locations between Friday and other working days during the evening rush hour and overall distribution.

**Figure 6:**
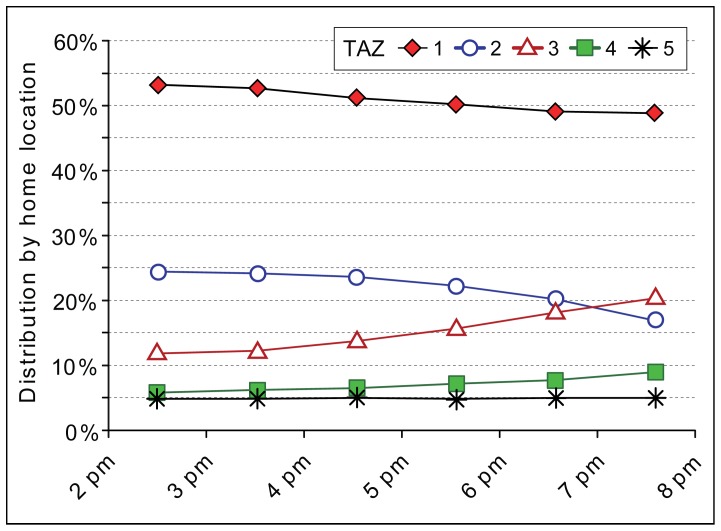
Hourly variance in the distribution of road users by home locations. **T**he distribution of home locations located in five transport analysis zones (TAZ, see Figure 2) indicates the average shares flow from Monday to Thursday.

The composition of road users’ home locations changes from the afternoon towards the evening (2:00 pm –8:00 pm). The share of suburbanites who use the studied road most probably on a daily basis (TAZ 3) start to increase and within 6 hours their share in traffic flow increases 71 percent as their distribution among road users has reached a level of 20.3% (Figure 6). On the contrary, the share of road users living in other parts of the Tallinn FUR and in Tallinn steadily decreases towards the evening.

**Figure 7:**
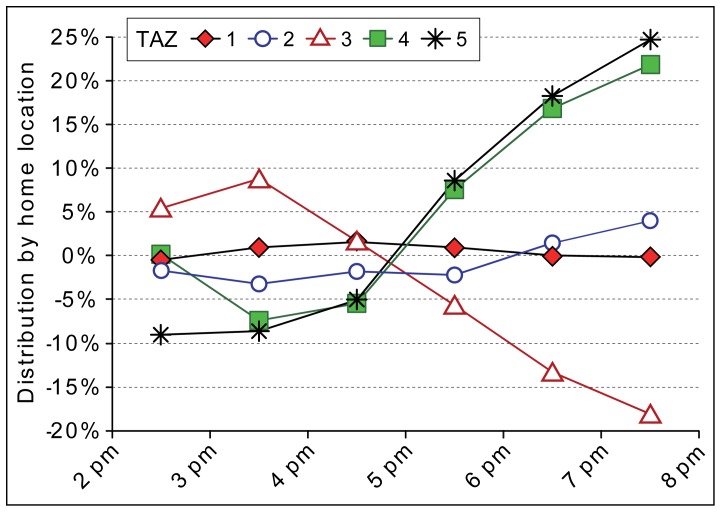
Difference in the distribution of road users by home location on Friday’s. The difference indicates to what extent the distribution of road users from each of the five transport analysis zones (TAZ, see Figure 2) on Friday’s is different compared to other working days.

The composition of road users on Friday evening compared to the temporal trend on other working days is different (Figure 7). Prior to evening rush hour traffic, the distribution of suburbanites living in the catchment area of the studied road section (TAZ 3) is 10 percent higher on Friday’s than on other working days. After the rush hour, their share decreased 17 percent compared to other working days at the same time. During the Friday evening rush hour, the share of road users living outside the Tallinn FUR (TAZ 4 & 5) begins to increase, although their overall distribution remains negligible (see Figure 5). This change on Friday might indicate that suburbanites who are most likely to use the studied road section on a daily basis (TAZ 3) are aware of traffic congestion on Friday evenings and thus try to avoid it to some extent by reallocating their travel behaviour to reschedule their movement to an earlier time. The increase in road users from outside the Tallinn FUR during and after the evening rush hour indicates weekend travel towards Tallinn.

**Figure 8:**
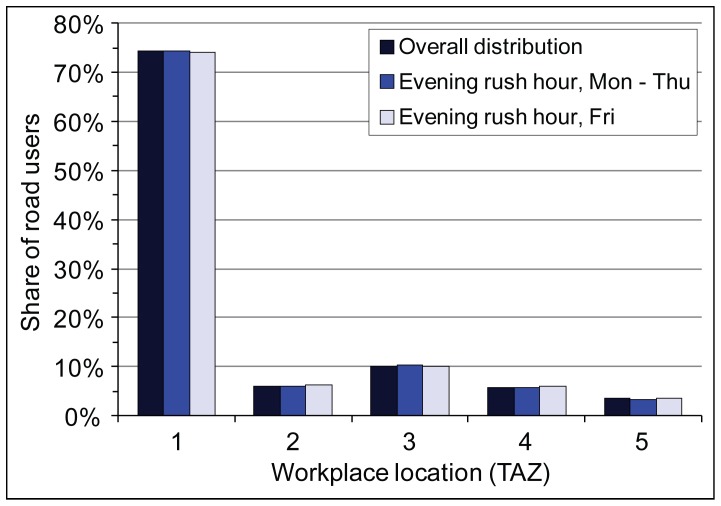
The overall distribution of road users by workplace locations. **T**he distribution of workplace locations located in five transport analysis zones (TAZ, see Figure 2) is shown separately for the annual overall distribution and during the evening rush hour on Friday’s and on other working days.

Not surprisingly, the majority of road users have workplaces (or educational facilities) located in Tallinn (Figure 8). Almost 74% of all road users’ workplaces are located in Tallinn (TAZ 1), whereas only 10% of workplaces are a part of the Tallinn FUR that is considered the catchment area of the studied road (TAZ 3), while 6% of workplaces remain elsewhere in the Tallinn FUR (TAZ 2). We found no differences in the composition of road users by their workplace locations during evening rush hour traffic, either on Friday or on other working days compared to annual overall distribution.

### Impact of Daily Commuting

**Figure 9:**
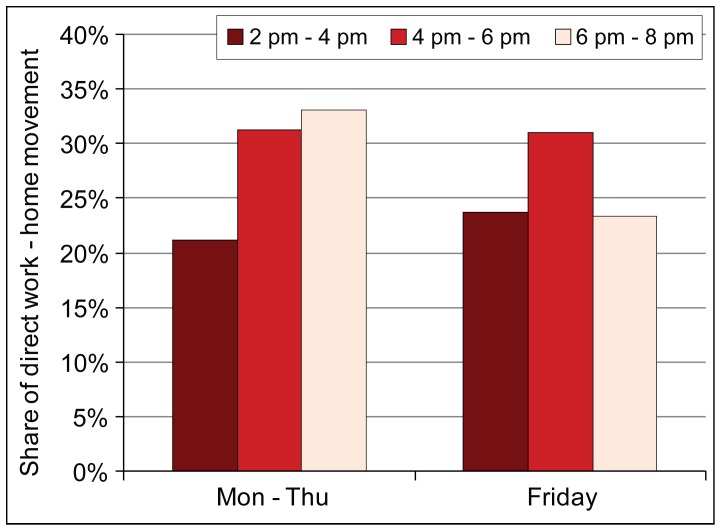
The share of daily workplace-home commuting from all traffic during the afternoon. The share is shown prior, during and after the evening rush hour, separately for Friday’s and other working days.

Because we are able to connect road users’ home and workplace location using the anchor point model, this enables us to assess their trip indication, e.g. daily commuting. The share of daily commuting which most probably is related to workplace-home movement is similar between Friday and other working days (Figure 9) while it comprises 31% of all trips in the studied road section. Accordingly, the majority of trips (69%) during the evening rush hour (4:00 pm –6:00 pm) are not related to daily commuting travel considering the relative locations of road users’ homes and workplaces from the studied road section. From Monday to Thursday the share of trips of daily commuters during the evening rush hour is significantly higher (+48%) than in the period prior (2:00–4:00 pm) and after the rush hour the share of trips of daily commuters continues to increase.

On Friday’s, however, the share of trips conducted by those daily commuters prior to the rush hour is slightly higher (+12%) than compared to the same time on other working days. Moreover, after the evening rush hour daily commuters make fewer trips relative to other working days while their share in all trips decreases 22 percent. These changes in time indicate that daily commuting trips are typically concentrated on the evening rush hour. However, on Friday the share of trips conducted by daily commuters might be higher in all traffic prior to the rush hour due to the fact that on a daily basis they are aware of Friday evening congestion and try to avoid it by reallocating their movement to home.

Daily commuting does not differ within movement directions or spatial context in the comparison between Friday’s and other working days (Table 2). The majority of daily commuting trips are related to the movement between Tallinn (TAZ 1) and the part of the Tallinn FUR that is considered to be the catchment area of the studied road (TAZ 3). We found equal movement volumes in both directions – both suburbanites moving from their workplaces in Tallinn to their suburban homes as well as road users from Tallinn who have workplaces located in the Tallinn FUR, respectively 10% and 11% of all movements during the rush hour. In total, 14 to 15% of all trips during the evening rush hour is created by suburbanites potentially commuting from the workplace to home on a daily basis. These results indicate that the share of daily commuters in the Friday evening rush hour remains equal to that on other working days, despite the overall increase in trips on Friday.

**Table 2:** Distribution of daily commuting during the evening rush hour.

Workplace – Home movement	Mon – Thu	Friday
FUR catchment area (TAZ 3) – Tallinn (TAZ 1)	11%	11%
Tallinn (TAZ 1) – FUR catchment area (TAZ 3)	10%	10%
FUR (TAZ 2) – FUR catchment area (TAZ 3)	5%	4%
FUR catchment area (TAZ 3) – FUR (TAZ 2)	1%	1%
Tallinn (TAZ 1) – Estonia, catchment area (TAZ 4)	2%	3%
Estonia, catchment area (TAZ 4) – Tallinn (TAZ 1)	2%	2%
Total	31%	31%

As mentioned above, the majority (69%) of road users’ trip purposes during the evening rush hour are not related to daily workplace-to-home movement and the composition by their origin remains similar both on Friday’s and on other working days (Table 3). A majority of these road users live in Tallinn and comprise in total about 37% of all road users during the evening rush hour. Some 20% of these road users are from the Tallinn FUR (TAZ 2) and 10% originate from outside the Tallinn FUR (TAZ 4 & 5).

**Table 3:** Distribution of non daily commuting by the origin during the evening rush hour.

Home location of road users	Mon – Thu	Friday
from Tallinn (TAZ 1)	37%	38%
from FUR (TAZ 2)	21%	20%
from TFUR, catchment area (TAZ 3)	1%	1%
From Estonia, catchment area (TAZ 4)	5%	5%
from Estonia, other (TAZ 5)	5%	5%
Total	69%	69%

### Final Destination of the Day

**Figure 10:**
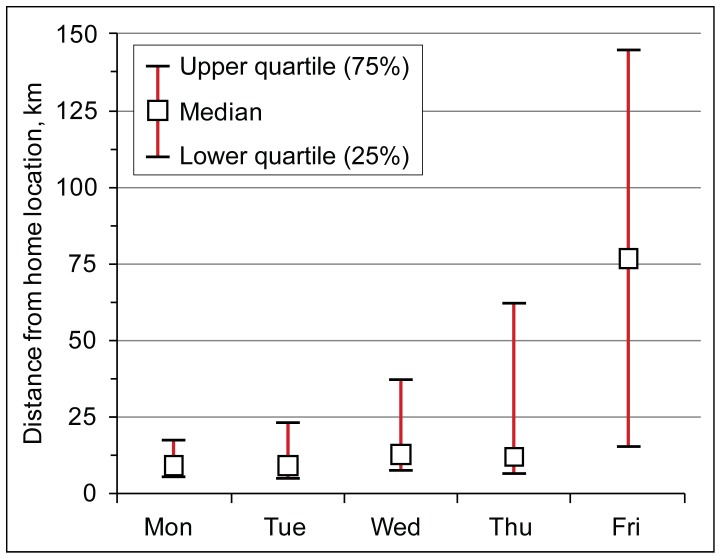
Distances between the home location and the last call activity of the day. Box-Whiskers plot indicates distances (km) for those road users who live in Tallinn and used the studied road section during the evening rush hour.

As the Friday evening rush hour is significantly higher compared to other working days, this could indicate that road users travel behaviour and trip purpose might be different on Friday’s (Figure 4). In addition, the above results show that during the evening rush hour, half of road users are people who live in Tallinn, whereas the majority of their trips are not related to daily commuting. Thus, we selected those rush hour road users who live in Tallinn and compared their final trip destinations of the day and the distance from their home location between working days. The distance between the home location and their final trip destination of the day shows a clear distinction between Friday’s and other working days (Figure 10). From Monday to Thursday, the distance between road users’ last call activity of the day and their home location remains in quite a similar range, i.e. 9–12 km from the home location. Moreover, only 6% of the last call activities of the day are located outside Tallinn. This suggests that during the evening rush hour road users from Tallinn who did not use the studied road section for daily commuting are nevertheless moving within their everyday activity space, and return to their home by the end of the day. In contrast, on Friday’s, the distance between the last call activity and the home location is far greater –77 km, whereas these distances are considerably dispersed. This is related to the fact that 13% of all road users living in Tallinn made their last call activity outside Tallinn, which is nearly three times as much as on other working days.

**Figure 11:**
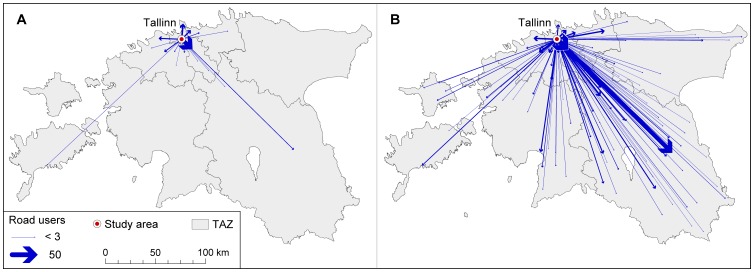
Spatial distribution of the last call activity of the day. Arrows on the map for Mon – Thu A) and for Fridays B) indicate the final destinations of the day which are located outside Tallinn for those road users who live in Tallinn and used the studied road section during the evening rush hour.

Friday’s difference from other working days is evident in the geographical aspect as well, and suggests that trip purposes have changed (Figure 11). While from Monday to Thursday the last call activities of the day, which are made outside Tallinn, are located predominantly in the Tallinn FUR (78%). On Friday, respectively, the figure is significantly lower (38%). Moreover, while from Monday to Thursday only 10% of all the last call activities of the day which are made outside Tallinn are located in central and southern Estonia (TAZ 4), yet on Friday this share increases threefold –39% of all the last call activities of the day that are made outside Tallinn are located in central and southern Estonia. The difference in the geographical distribution of the final destination of the day indicates that on Friday’s the purpose of movement during the evening rush hour is different than compared to other working days.

## Discussion

In this paper we put forward an alternative approach that uses automatically stored call activity data (i.e. call detail records) of mobile phones and converts it into space-time movement data of individuals in order to assess mobility of individuals and aggregated traffic flows. We showed that our approach is able to capture and study traffic flows and thus it substantially helps in putting together cost-effective origin-destination matrices for further analysis of traffic flows. However, we argue that the proposed method has a high potential addition to traditional methods, and could substitute large-scale questionnaires, on-road surveys or large-scale number plate detections to some extent. Yet, it does not help with aspects like transportation mode split or trip chaining, and therefore we still need classical traffic counters for calibrations.

Our approach is theoretically applicable in real time and can be considered suitable in the framework of ITS. For a real-time perspective, all necessary requirements are met today – suitable datasets from the mobile network operator, legislation (for example in EU), computational power and analytical know-how to provide valuable information on traffic. Already, based on handover data of mobile phones and Erlang data from the mobile network, traffic flows are extracted and monitored along with the assessment of congestion levels in real-time [64]. However, limiting factors on the one hand are debates regarding privacy and surveillance issues, and thus, unwillingness of the network operators to provide data in order to avoid involvement in these debates. On the other hand, network operators have not yet realized that mobile phone usage-related data has important secondary value, which embody the potential for profitable business [67].

The driving motivation for our research was to determine the influence of suburbanites on rush hour traffic. It is precisely rush hour traffic congestions that are, often without justification, associated with suburban commuters. Suburbanisation certainly has a significant influence on traffic intensity, yet we found that in a working day only one third of road users’ trips during the evening rush hour are related to daily commuting, whereas only one sixth of trips are conducted by commuting suburbanites. This study indicates that public opinion in Estonia is exaggerating the ‘blame’ against commuters or suburbanisation for being the cause of worsening traffic conditions.

Further, one of the main purposes of the academic dialogue concerning the relationship between transportation and land use has been to question whether land use should be regulated by policies in order to steer transportation demand [1], [28]. To this end, at least in the case of the studied road section in Tallinn, if land use regulations were to be applied in order to reduce the impact of suburban commuters on a traffic flow, it would affect no more than one third of trips during the evening rush hour. No matter how well land use regulations and spatial planning is applied, or how well society is aware of wasteful transportation use (e.g. excessive commuting [90]), it may not reduce commuters transportation demand enough as long as people are individualistic and more interested in a comfortable lifestyle, e.g. [40]. Nonetheless, during the evening rush hour the share of trips by daily commuters increases compared to prior the rush hour as workplace-to-home related movement is concentrated on that period. In other words, we can conclude that commuting suburbanites do have an impact on rush hour traffic conditions.

Results show that two thirds of trips during the evening rush hour are not solely of daily workplace-home related traffic. This refers to increasing overall mobility of individuals and coincides with previous studies finding leisure activities as a predominant travel purpose [44]. The Friday evening rush hour, as the worst traffic congestion of the week in the studied road section, is caused predominantly by increasing overall individual mobility in the society – both daily commuters as well as other road users are more mobile than on other working days due to the start of the weekend and leisure time activities. In addition, road users’ final destination of the day indicates that on Friday’s the purpose of movement is different than on other working days, and is related more to weekend travel behaviour that takes place outside their everyday activity space. To some extent this is related to visiting relatives and the fact that more and more Estonian people own second homes [91].

### Conclusion

In this study we developed a method based on call detail records of mobile phones in order to assess the composition of actual traffic flow. We sought to shed light on how suburbanite commuters influence traffic flow by conducting an empirical study on evening rush hour traffic in one of the main arterial highway section near the city limits of Tallinn, Estonia. We found daily commuting trips influencing transportation demand by amplifying during the evening rush hour period, although suburban commuters’ trips have a modest impact on the evening rush hour traffic. The dominant share of trips by all road users is not related to the workplace-home movement.

The developed method enables the possibility to study geographical distribution and temporal variability of trips in a traffic flow. When applying such data and methodology, it is easy to detect road users in a given area and certain timeframe (for example during the rush hour) and link their origin and destination of movement with their trip indication. With traditional methods it would be complicated and expensive to obtain this information. At the moment, the two main shortcomings of this approach are that we cannot reveal the actual purpose behind the movement, nor can we reveal the modal split for road users. In addition, one must always address privacy issues related to the data used. This study, hence, showed that this approach can cost-effectively provide additional valuable information for traffic studies, particularly in terms of the composition of road users in spatial and temporal contexts. Moreover, this method could be adopted as one of the real-time traffic monitoring tools for ITS in the near future.

Next steps for this research would be the real-time applicability along with the comparison of the traffic composition between different road sections as promising avenues for further research of implementing mobile phone data.

## Supporting Information

Text S1 Description of study area.
(PDF)
