# Supplementary material for: Mobile Phones in a Traffic Flow: A Geographical Perspective to Evening Rush Hour Traffic Analysis Using Call Detail Records
Source: PLoS One. 2012 Nov 14;7(11):e49171. doi: 10.1371/journal.pone.0049171 (PMC3498329; doi:10.1371/journal.pone.0049171)
Supplement: Text S1 — Description of study area. (PDF) [file pone.0049171.s001.pdf]

Text S1. Description of study area

Tallinn functional urban region (FUR) is a monocentric urban system comprising relatively compact core city of Tallinn, with 2520 inhabitants/km^2 and sparsely populated FUR (33 inhabitants/km^2). Most populated municipalities other than Tallinn (400,000 inhabitants) in the Tallinn FUR have less than 20,000 inhabitants [1]. The study area underwent two major changes within the last two decades, after the restoration of Estonian independence in 1991.

First, the Tallinn FUR witnessed relatively rapid urban processes including a vast suburbanisation. Suburbanisation process in Estonia, however, differs from a western, classical suburbanisation process because a remarkable share of residential space is being built as apartment buildings [2]. The suburban lifestyle is highly dependent on the regional centre, i.e. workplace, school, maintenance services and leisure time activities are directly related to the city centre of Tallinn [3]. Since 2000, workplaces and services have begun to relocate from Tallinn to the neighbouring municipalities in FUR.

Secondly, a remarkable motorisation has occurred. The number of registered vehicles in Estonia has more than doubled within two decades and in 2009 there were 407 passenger vehicles per thousand inhabitants [4]. Still, the figure is 14% lower than the average of the 27 European Union countries. Therefore it is very likely that the motorisation trend in Estonia will continue in the near future as society develops.

References

1. Statistics Estonia (2011) CC41: Telecommunication services. Available: http://www.stat.ee. Accessed 13 May 2011.

2. Tammaru T, Leetmaa K, Silm S, Ahas R (2009) Temporal and Spatial Dynamics of the New Residential Areas around Tallinn. Eur Plann Stud 17: 423-439.

3. Ahas R, Silm S, Järv O, Saluveer E, Tiru M (2010) Using Mobile Positioning Data to Model Locations Meaningful to Users of Mobile Phones. J Urban Technol 17: 3-27.

4. European Commission (2012) EU transport in figures: Statistical pocketbook 2012. Luxembourg: Publications office of the European Union. 132 p. Available: http://ec.europa.eu/transport/facts-fundings/statistics/doc/2012/pocketbook2012.pdf. Accessed 22 September 2012.
